# Inverse probability weighting and doubly robust methods in correcting the effects of non-response in the reimbursed medication and self-reported turnout estimates in the ATH survey

**DOI:** 10.1186/1471-2458-14-1150

**Published:** 2014-11-06

**Authors:** Tommi Härkänen, Risto Kaikkonen, Esa Virtala, Seppo Koskinen

**Affiliations:** Department of Health, Functional Capacity and Welfare National Institute for Health and Welfare (THL), P.O. Box 30, FI-00271 Helsinki, Finland

**Keywords:** Missing data, Population survey, Register data, Model selection, Inverse probability weighting, Doubly robust methods

## Abstract

**Background:**

To assess the nonresponse rates in a questionnaire survey with respect to administrative register data, and to correct the bias statistically.

**Methods:**

The Finnish *Regional Health and Well-being Study (ATH)* in 2010 was based on a national sample and several regional samples. Missing data analysis was based on socio-demographic register data covering the whole sample. Inverse probability weighting (IPW) and doubly robust (DR) methods were estimated using the logistic regression model, which was selected using the Bayesian information criteria. The crude, weighted and true self-reported turnout in the 2008 municipal election and prevalences of entitlements to specially reimbursed medication, and the crude and weighted body mass index (BMI) means were compared.

**Results:**

The IPW method appeared to remove a relatively large proportion of the bias compared to the crude prevalence estimates of the turnout and the entitlements to specially reimbursed medication. Several demographic factors were shown to be associated with missing data, but few interactions were found.

**Conclusions:**

Our results suggest that the IPW method can improve the accuracy of results of a population survey, and the model selection provides insight into the structure of missing data. However, health-related missing data mechanisms are beyond the scope of statistical methods, which mainly rely on socio-demographic information to correct the results.

## Background

It has been widely acknowledged that non-response in population surveys is an increasing problem [1–4]. The mechanisms of non-response are often related to socio-demographic factors (e.g. gender, age, education, marital status), which can be found in administrative records. In these cases, where the dropout probability depends on observed factors such as those obtained from administrative records, the mechanism can be considered missing-at-random (MAR), and any resulting bias can be corrected. For example, the non-response rate has been high in the groups of young single men with low education [5]. It is unclear if the participants in this group (or other groups) can fully represent the non-respondents. The mechanisms are often related to health, as has been demonstrated in studies on mortality [6] and hospital admission rates [7]. Lifestyle factors also have an impact on dropout rates, as demonstrated in longitudinal population studies where, for example, smokers have been shown to be more prone to drop out from the follow-up [8]. In these cases, however, the mechanism depends (at least partly) on unobserved factors and is called not-missing-at-random (NMAR). The results based on the observed data may be biased [9–11]. This type of bias is virtually impossible to correct without some external information on the NMAR mechanism, although research has been conducted comparing respondents of a baseline survey with non-respondents who were later interviewed by telephone [12]. Earlier studies have analyzed non-participation mechanisms [13–15], but there seem to be few recent papers that seek to adjust for the effects of non-response using register data on health outcomes for the full sample.

In Finland, we have an excellent opportunity to link the survey data with administrative registers individually, which allows us to obtain the full-sample estimates and to use these as the true results. A comparison of methods for handling the effects of missing data is on a solid ground if the outcome variable can be observed both for the participants and nonparticipants as in our case. The most important registers used in the present study are maintained by the Population Register Centre (VRK), Statistics Finland (TK) and the Social Insurance Institution (Kela). The study sample was drawn by VRK, which also provided the basic demographic information as well as tabulations on the population sizes. TK provided information on occupation and level of education. Kela provided information on reimbursement of medicine costs contained in the Prescription Register. These registers cover the entire Finnish population, and they are considered to have good accuracy, reliability and validity [16–18].

In this paper, we demonstrate the effects of non-response and statistical methods based on inverse probability weighting (IPW) and doubly robust (DR) methods to correct the effects of non-response. The weights were estimated using socio-demographic register-based variables. The weighted prevalence estimates of the reimbursement of medicine costs and turnout for the municipal election were compared with the corresponding and actual reimbursement and turnout prevalences in the sample based on the register data.

## Methods

### Data

The *Regional Health and Well-being Study (ATH)* (n = 31,000) was conducted in three areas in Finland in 2010: Turku, the Oulu region and the Kainuu region. An additional nationally representative sample was also collected.

In Finland, local authorities have a statutory obligation to monitor the health and well-being of population groups. National registries provide municipal-level information on the population’s demographic characteristics and diseases treated by the public health care system. However, there is no municipal-level information on self-assessed health and well-being or the need for health and social services. The ATH survey aims to provide local information on the latter phenomena.

ATH 2010 was a questionnaire survey aimed at the population aged 20 years or over. The respondents could either return the questionnaire by mail or reply to the questions online. Three versions of the questionnaire were prepared (for the age groups 20–54, 55–74 and 75+) in four languages – Finnish, Swedish, Russian and English. Information on the respondent’s principal language was obtained from VRK, and this information was used in selecting the language of the questionnaire that was mailed to the respondent. The ATH survey was approved by the Ethics Committee of National Institute for Health and Welfare (THL). Participation was voluntary, and subjects were informed of register linkages. Participants were thus considered to have given their informed consent to the linking of health and well-being registers.

### Sampling design

The stratified random sampling design was applied in order to provide enough statistical power to estimate the differences between the small areas accurately. The 15 strata (and corresponding sample sizes) were defined by geographical areas as described here. The City of Turku was divided into nine areas (sample size 1,000 per area) and the province of Kainuu into three areas (3,000 per area). Two sub-regions of the province of Northern Ostrobothnia (3,000 in the southern Oulu region and 5,000 in the Oulu region) were also included. In addition, one nationally representative random sample (5,000) was drawn from the entire population of Finland. Furthermore, each geographical stratum was subdivided by gender and the age groups 20 to 74 and 75 to 99. The sampling fractions ranged between 0.1% (younger age groups in the whole of Finland) and 48.6% (older women in the Upper Kainuu region). The population sizes were rather small in most of the strata, and thus the sampling was without replacement (WOR).

### Variables used in the analyses

Age, gender, marital status dichotomized as married or unmarried, and first language were derived from VRK register data. The level of education was derived from TK register data and categorized as primary or unknown, secondary and tertiary level education.

The entitlements to reimbursement of medicine costs during 2010 were coded as binary variables and obtained from the Prescription Register of Kela, which has been shown to have good accuracy and coverage in the context of Finnish registries [19–21].

The turnout percentage for the 2008 municipal election in various areas of Finland was obtained from the official website. All citizens of Finland, the EU and the Nordic countries aged 18 years or above and resident in Finland were eligible to vote in their home municipality. At the time of the ATH study, these subjects were 20 years old or more, which matches the age range of the ATH study. The self-reported turnout in the ATH study was based on the question “Did you vote in the previous municipal election?”

The body mass index (BMI) was calculated by dividing the self-reported weight (kg) by the squared self-reported height (m).

### Statistical methods

There were three sources of missing data. Firstly, only part of the finite population was selected in the sample (missing by design). The sampling fraction was relatively high (up to 48.6%) in some strata, and thus finite population correction (FPC) was incorporated in the analyses. Secondly, not all individuals in the sample participated (unit-nonresponse). Thirdly, some participants did not respond to individual questions (item-nonresponse).

The effects of missing data were accounted for using inverse probability weighting (IPW) [10, 22]. The unit non-response probabilities were estimated using a logistic regression model. The register-based variables age, gender, marital status, language, education, occupation and geographical area were used as independent variables and the response indicator as the outcome variable. The model selection was conducted using the Bayesian information criterion [23], which is suitable for comparing several (different) models applied to the same data set and accounts for possibly large sample sizes and avoids overly complicated models [24]. The family of compared models contained the main effect models and the models with main effects and one or two first-order interactions. Also, propensity score methods [25] with weights calibrated by the generalized boosted model [26] (GBM) were applied to assess the potential benefits of having a more saturated weighting model. So-called doubly robust methods [27] are often based on an analysis model and a weighting model, in which a (linear) model is used for the outcome variable and a (logistic) regression model for the participation indicator, respectively. It has been demonstrated that, in order to obtain unbiased results, it is sufficient if one of the aforementioned models is correct. This methodology has also been applied when adjusting for missing data by Wirth et al. [28], and we have directly applied their method and program code written in SAS (which we converted into R) in this work. We have used all first-order interactions of the covariates except language, which was entered into the model as a main effect, for both the outcome and weighting models. As there were only 501 non-respondents for the BMI questions (1.6% of the sample or 3% of the participants) and 430 for the self-reported turnout questions, we decided not to elaborate the IPW in order to account for the item-nonresponse. The different sampling fractions were accounted for by calibrating the weights so that the sum of the expansion weights was proportional to the population size in each stratum.

We report the 95% confidence intervals (CI) of the statistics.

The R software [29] and the survey package [30] were used in the analyses. The GBM was implemented in the twang package [31].

## Results

The sampling percentages varied considerably across geographical strata (Table 1). In continental Finland, only 0.1% in the age group 20 to 74 and 0.2% in the age group 75 to 99 were sampled. In the regional strata, the sampling percentages were much higher, the highest being in Upper Kainuu (20% and 48.6%, respectively) and the Maaria-Paattinen region of the city of Turku (21% and 43.5%, respectively).

**Table 1:** **Population and sample sizes in the strata defined by area, gender and age group**

Age group:	20-74					75-99				
Gender:	Women		Men		All	Women		Men		All
Area	N ^*^	n ^†^	N ^*^	n ^†^	% ^‡^	N ^*^	n ^†^	N ^*^	n ^†^	% ^‡^
Continental Finland	1,824,368	1,943	1,805,153	2,057	0.1	257,680	638	145,886	362	0.2
Kainuu										
Kajaani area	14,737	1,117	14,929	1,229	7.9	2,344	422	1,287	232	18.0
Kuhmo-Sotkamo	6,665	1,015	7,261	1,206	15.9	1,254	469	827	310	37.4
Upper Kainuu	4,903	916	5,560	1,177	20.0	1,141	555	725	352	48.6
Ostrobothnia										
Southern Oulu region	16,629	1,074	17,654	1,236	6.7	2,769	428	1,695	262	15.5
Oulu region	74,303	2,090	74,981	2,207	2.9	7,232	448	4,119	255	6.2
Turku										
Keskusta	20,373	385	17,710	369	2.0	3,675	170	1,632	76	4.6
Hirvensalo-Kakskerta	2,532	483	2,285	441	19.2	117	46	74	30	39.8
Skanssi-Uittamo	8,591	380	7,291	352	4.6	1,615	176	843	92	10.9
Varissuo-Lauste	6,680	438	6,027	416	6.7	676	98	328	48	14.5
Nummi-Halinen	7,355	427	7,368	445	5.9	638	82	368	46	12.7
Runosmäki-Raunistula	5,825	398	5,287	388	7.1	872	140	464	74	16.0
Länsikeskus	7,111	386	6,562	386	5.6	1,150	150	602	78	13.0
Pansio-Jyrkkälä	3,364	422	3,422	450	12.8	301	84	160	44	27.8
Maaria-Paattinen	2,185	454	2,182	462	21.0	114	50	79	34	43.5

^*^Population size.

^†^Sample size.

^‡^Sampling proportion (%) in the age group.

The unadjusted distributions of participation rates showed considerable variation with respect to both gender and several socio-demographic variables obtained from administrative registers (Table 2). The lowest participation rates were in the group comprising the youngest men. The participation rates increased with age, and in the oldest age groups the rates for men were at the same level as for women, among whom the rates varied much less. The participation rates in the stratum of continental Finland were 51.6% for women and 39.7% for men.

**Table 2:** **Sample sizes and participation rates in subgroups of the sample**

		Women		Men	
Variable	Group	n ^*^	Participation (%) ^†^	n ^*^	Participation (%) ^†^
Age	20-24	1,063	40.4	1,158	22.5
	25-34	2,208	43.9	2,486	27.0
	35-44	2,013	45.0	2,216	31.2
	45-54	2,435	53.7	2,720	39.5
	55-64	2,547	62.2	2,746	52.2
	65-74	1,662	67.1	1,495	60.4
	75-84	2,940	57.4	1,907	60.6
	85-99	1,016	43.2	388	43.8
Area	Continental Finland	2,581	51.6	2,419	39.7
	Kainuu				
	Kajaani area	1,539	56.5	1,461	46.0
	Kuhmo-Sotkamo	1,484	57.2	1,516	45.3
	Upper Kainuu	1,471	59.6	1,529	47.7
	Ostrobothnia				
	Southern Oulu region	1,502	53.7	1,498	40.7
	Oulu region	2,538	50.9	2,462	39.2
	Turku				
	Keskusta	555	52.4	445	43.4
	Hirvensalo-Kakskerta	529	45.0	471	37.2
	Skanssi-Uittamo	556	55.8	444	48.0
	Varissuo-Lauste	536	52.6	464	36.6
	Nummi-Halinen	509	50.3	491	37.7
	Runosmäki-Raunistula	538	53.5	462	43.3
	Länsikeskus	536	55.2	464	47.2
	Pansio-Jyrkkälä	506	42.5	494	36.0
	Maaria-Paattinen	504	47.2	496	41.1
Married	Unmarried	8,729	49.8	7,256	32.9
	Married	7,155	57.2	7,860	50.6
Language	Other	535	47.9	473	40.4
	Finnish	15,349	53.3	14,643	42.1
Education	Basic or unknown	5,561	48.4	4,899	41.9
	Vocational	5,954	51.8	6,624	37.1
	Lowest academic	1,843	62.8	1,275	56.2
	Lower academic	1,332	57.7	1,223	47.4
	Higher academic	1,085	61.8	930	50.0
	Researcher	109	60.6	165	50.9

^*^Sample size.

^†^Participation rate.

The interactions of gender and age and of gender and marital status in addition to the main effects of age (categorical), gender, area, marital status, language and education were important predictors of non-response according to the model selection procedure based on the family of different main effect and first-order interaction logistic regression models and the BIC statistic (Table 3). Occupation appeared to have no significance when education was included in the model (data not shown). The root mean squared errors (RMSE) of the predicted probability of response with respect to the observed response were 0.476 and 0.472 for the IPW and GBM, respectively.

**Table 3:** **The best models selected using the Bayesian information criteria (BIC)**

Model ^*^	BIC ^†^
Main effects + Gender*Age + Gender*Married	40356
Main effects + Gender*Married	40361
Main effects + Gender*Married + Gender*Education	40365
Main effects + Age*Gender	40367
Main effects + Gender*Married + Married*Language	40369
Main effects + Gender*Married + Gender*Language	40371
Main effects + Age*Gender + Married*Language	40376
Main effects + Age*Gender + Gender*Language	40377
Main effects	40379

^*^The specification of independent effects of the logistic regression models.

^†^The Bayesian information criteria.

Main effects correspond to age (categorical), gender, area, marriage status, language and education. Lower values of BIC correspond to parsimonious models that predict the nonresponse well.

The adjusted participation rate was lowest in the group of the youngest men (OR = 0.62) compared to the age group 45 to 54 (Table 4). The participation rates increased with age. In the age group 75 to 84, the OR was 3.02. In the age group 45 to 54, women had a higher participation rate (OR = 1.90), but in the oldest age groups this difference decreased (interaction OR = 0.60 for men and 0.64 for women in the age group 75 or older). In comparison to Continental Finland, in the province of Kainuu the participation rates were higher, while they were at the same level in the province of Ostrobothnia and the city of Turku. In the city of Turku, the lowest participation rates were in the wealthiest area (Hirvensalo-Kakskerta, OR = 0.75) and the least wealthy area (Pansio-Jyrkkälä, OR = 0.87). Married men had a higher participation rate than unmarried men (OR = 1.53). Subjects speaking Finnish as their principal language had a slightly higher participation rate than the rest of the sample (OR = 1.19). In the groups with higher education, the participation rates were higher than in the groups with basic or unknown (OR = 0.37) or vocational (OR = 0.59) education.

**Table 4:** **The odds ratio estimates of the response model with 95% confidence intervals**

	OR ^*^	(95% CI) ^†^		OR ^*^	(95% CI) ^†^
**Intercept**	0.64	(0.53 - 0.77)	**Gender**		
**Age group**			Men	1.00	
20-24	0.62	(0.53 - 0.73)	Women	1.90	(1.67 - 2.15)
25-34	0.62	(0.55 - 0.7)	**Marriage status**		
35-44	0.69	(0.61 - 0.78)	Not married	1.00	
45-54	1.00		Married	1.53	(1.43 - 1.65)
55-64	1.78	(1.59 - 1.98)	**Language**		
65-74	2.73	(2.38 - 3.12)	Other	1.00	
75-84	3.02	(2.66 - 3.43)	Finnish	1.19	(1.04 - 1.37)
85-99	1.71	(1.37 - 2.14)	**Education**		
**Area**			Basic or unknown	0.37	(0.33 - 0.42)
Continental Finland	1.00		Vocational	0.59	(0.54 - 0.66)
Kainuu			Lowest academic	0.89	(0.79 - 1.00)
Kajaani area	1.24	(1.12 - 1.36)	Lower academic	0.89	(0.78 - 1.00)
Kuhmo-Sotkamo	1.19	(1.08 - 1.31)	Higher academic	1.00	
Upper Kainuu	1.33	(1.21 - 1.46)	Researcher	0.87	(0.67 - 1.14)
Ostrobothnia					
Southern Oulu region	1.06	(0.97 - 1.17)	**Interaction between age and gender**		
Oulu region	0.99	(0.92 - 1.08)	20-24 Women	1.22	(0.98 - 1.53)
Turku			25-34 Women	1.12	(0.95 - 1.33)
Keskusta	1.15	(1.00 - 1.33)	35-44 Women	0.99	(0.84 - 1.17)
Hirvensalo-Kakskerta	0.75	(0.65 - 0.87)	45-54 Women	1.00	
Skanssi-Uittamo	1.22	(1.06 - 1.41)	55-64 Women	0.92	(0.78 - 1.08)
Varissuo-Lauste	1.03	(0.89 - 1.19)	65-74 Women	0.88	(0.73 - 1.06)
Nummi-Halinen	1.04	(0.90 - 1.21)	75-84 Women	0.6	(0.51 - 0.71)
Runosmäki-Raunistula	1.13	(0.98 - 1.30)	85-99 Women	0.64	(0.49 - 0.84)
Länsikeskus	1.18	(1.03 - 1.37)	**Interaction between gender and marital status**		
Pansio-Jyrkkälä	0.87	(0.75 - 1.01)	Women Married	0.79	(0.72 - 0.87)
Maaria-Paattinen	1.06	(0.92 - 1.22)			

^*^Odds Ratio.

^†^95% confidence interval.

The true turnout percentages of the 2008 municipal election were much lower than the estimates based on the self-reported turnout (Table 5). In continental Finland, the true percentage was 61.3%, and after adding the self-reporting bias the percentage, which was based on [32] and comparable to our estimates, was 70.7% whereas the crude, uncorrected estimate was 78.2% (CI 76.5-80.0). The weighting improved the estimate, but in comparison to the true turnout this corrected estimate was still slightly higher, 72.9%; however, the confidence interval (CI 70.6-75.1) contains the bias-added turnout estimate. The corresponding differences were significant in the Turku and Kainuu areas but not in Ostrobothnia.

**Table 5:** **The turnout percentages of the 2008 municipal election in different areas of Finland**

	True results		Reported by respondents in ATH					
Area	True ^*^	Self-reporting bias added ^#^	Crude ^†^	95% CI ^§^		Weighted ^‡^	95% CI ^§^	
Continental Finland	61.3	70.7	78.2	(76.5	80.0)	72.9	(70.6	75.1)
Turku	58.6	68.8	77.3	(76.0	78.6)	72.9	(71.0	74.8)
Kainuu	53.7	65.2	71.9	(70.6	73.3)	68.5	(66.9	70.1)
Southern Oulu region	63.5	72.4	78.0	(75.8	80.3)	73.5	(70.8	76.1)
Oulu region	58.3	68.6	74.6	(72.8	76.5)	69.6	(67.3	71.8)

^*^True turnout percent by the Election Unit at the Ministry of Justice of Finland.

^#^Self-reporting bias added using Granberg and Holmberg [32]; 99% of the voters and 26% of the non-voters said that they voted.

^†^Crude turnout percent.

^‡^Weighted turnout percent.

^§^95% confidence interval.

There were some differences between the crude and weighted estimates of BMI in different areas in the subpopulation of people aged 20 to 59 with basic or unknown education (Table 6). In Turku, the weighted estimates were 0.66 BMI points lower than the crude averages. The crude prevalence of obesity (BMI value at least 30) was 26%, but the weighted prevalence was 19% in Turku. In other areas, the differences appeared to be much smaller.

**Table 6:** **Subset of individuals aged 20 to 59 years with basic or unknown education in different areas of Finland**

Area	Crude mean	(95% CI) ^*^		Weighted mean ^§^	(95% CI) ^*§^		Difference
Continental Finland	27.8	26.9	28.7	27.7	26.8	28.7	−0.1
Kainuu, Kajaani area	27.0	25.9	28.2	27.3	26.2	28.4	0.3
Kainuu, Kuhmo-Sotkamo	28.0	26.8	29.2	28.1	26.9	29.3	0.1
Kainuu, Upper Kainuu	26.3	25.4	27.3	26.2	25.2	27.1	−0.1
Southern Oulu region	27.1	25.9	28.2	27.0	25.8	28.2	−0.1
Oulu region	26.5	25.7	27.4	26.3	25.5	27.2	−0.2
Turku	27.6	26.9	28.4	27.0	26.3	27.7	−0.6

^*^95% confidence interval.

^§^Sampling design (strata, finite population correction, and sampling probabilities and non-response using weights) was accounted for.

Participants aged 20 to 59 years with basic or unknown education in different areas of Finland.

The FPC had some influence on results where the sampling fraction was large. In Kainuu, the standard error of BMI mean was 0.263 without FPC and 0.235 with FPC in the age group 75 to 99. In the Upper Kainuu region, the corresponding figures were 0.219 and 0.157. In other areas or age groups, FPC had virtually no influence on the results.

The IP weights appeared to remove most of the bias caused by nonresponse in the various subpopulations (Table 7). The true prevalence of entitlement to specially reimbursed medication for any chronic disease was 29.6% for men and 32.2% for women, whereas the sampling weighted (SW) estimates were 38.3% and 34.1%, respectively. The largest differences were related to men. Antipsychotic medication prevalences were underestimated by 1 percentage point in the Oulu region, whereas in the other regions the differences were much smaller; the prevalence estimates of other neurological medication were close to the true values in all areas. The performance of the doubly robust (DR) method for any chronic disease was close to that of the IPW method. The RMSE of the sampling weighted, IPW and DR were 4.36, 1.19 and 1.75, respectively. Thus, the SW estimates were much more biased than the IPW and DR estimates. However, in the disease-specific prevalences the three methods performed almost the same, and the RMSE ranged between 0.32 (other neurological disease estimates based on IPW) and 0.88 (diabetes estimates based on SW).

**Table 7:** **Prevalence of entitlement to specially reimbursed medication for any disease and for selected chronic diseases**

Subset	True	Crude ^§^	95% CI ^*^		SW ^#^	95% CI		IPW ^†^	95% CI ^*^		DR ^‡^	95% CI ^*^	
**Diabetes**													
Continental Finland	**6**	**7.6**	6.5	8.7	**6.6**	5.6	7.6	**6.2**	5.2	7.2	**6.4**	5.6	7.1
Turku	**4.6**	**6.7**	6	7.5	**5.9**	5.1	6.7	**5.4**	4.6	6.2	**5.3**	4.8	5.8
Kainuu	**6.6**	**8.9**	8	9.7	**7.3**	6.5	8.1	**6.8**	6	7.5	**6.7**	6.2	7.3
Southern Oulu region	**6.9**	**9.3**	7.7	10.8	**7.5**	6.2	8.8	**6.5**	5.4	7.7	**6.8**	5.9	7.6
Oulu region	**4.4**	**5.8**	4.9	6.8	**4.9**	4.1	5.8	**4.2**	3.4	5	**4.4**	3.9	5
Men	**6.7**	**7.2**	6.6	7.7	**8.2**	6.7	9.8	**5.1**	4.5	5.7	**7.3**	6.9	7.8
Women	**5.3**	**8.2**	7.5	8.8	**5.3**	4.2	6.4	**5.6**	5	6.2	**5.2**	4.8	5.6
Age group 20-54	**2.6**	**2.5**	2.1	2.9	**3.2**	2.2	4.1	**2.3**	1.9	2.8	**3.8**	3.4	4.1
Age group 55-74	**10.2**	**9.6**	8.8	10.4	**8.8**	7	10.6	**9.3**	8.3	10.2	**9.2**	8.5	9.8
Age group 75-99	**14.4**	**14.5**	13.3	15.7	**14.3**	11.6	17.1	**14**	12.6	15.3	**14.3**	13.4	15.2
**Other neurological diseases**													
Continental Finland	**2.2**	**2.4**	1.8	3.1	**2**	1.4	2.5	**2**	1.4	2.7	**2**	1.6	2.5
Turku	**2**	**1.9**	1.5	2.3	**1.8**	1.3	2.2	**1.6**	1.2	2.1	**1.6**	1.4	1.9
Kainuu	**2.7**	**2.5**	2.1	3	**2.3**	1.8	2.8	**2.4**	1.9	2.9	**2.5**	2.1	2.9
Southern Oulu region	**2.3**	**2.4**	1.6	3.2	**2.1**	1.4	2.9	**2.1**	1.3	2.8	**2**	1.5	2.5
Oulu region	**2.3**	**2.6**	2	3.3	**2.3**	1.7	2.9	**2.3**	1.6	2.9	**2.3**	1.9	2.8
Men	**2.5**	**2.3**	2	2.7	**2.5**	1.6	3.4	**1.9**	1.5	2.3	**2.2**	1.9	2.5
Women	**1.9**	**2.4**	2	2.7	**1.6**	1	2.2	**2.2**	1.8	2.6	**1.8**	1.6	2.1
Age group 20-54	**1.6**	**1.5**	1.2	1.8	**1.4**	0.7	2	**1.5**	1.2	1.9	**1.6**	1.4	1.8
Age group 55-74	**2.6**	**2.6**	2.2	3	**1.9**	1	2.7	**2.6**	2.1	3.1	**1.9**	1.6	2.2
Age group 75-99	**4.5**	**3.6**	2.9	4.2	**5.3**	3.5	7	**4**	3.2	4.8	**5.2**	4.6	5.8
**Psychosis**													
Continental Finland	**2.2**	**2**	1.4	2.6	**2**	1.4	2.6	**2.3**	1.5	3.1	**2.5**	1.8	3.2
Turku	**1.5**	**1.2**	0.9	1.6	**1.2**	0.8	1.6	**1.3**	0.9	1.7	**1.3**	1	1.5
Kainuu	**2.7**	**2.2**	1.8	2.6	**2.2**	1.8	2.7	**2.5**	1.9	3.1	**2.6**	2.2	3.1
Southern Oulu region	**2.8**	**2**	1.3	2.7	**2**	1.2	2.7	**1.9**	1.2	2.6	**1.9**	1.4	2.5
Oulu region	**1.9**	**1.1**	0.7	1.5	**1**	0.6	1.4	**1**	0.6	1.5	**1**	0.7	1.3
Men	**2.1**	**1.7**	1.4	2	**2.4**	1.4	3.3	**1.6**	1.2	1.9	**2.9**	2.5	3.3
Women	**2.3**	**1.7**	1.4	2	**1.7**	1	2.3	**1.6**	1.2	1.9	**1.6**	1.4	1.8
Age group 20-54	**1.8**	**1.5**	1.2	1.8	**1.9**	1.1	2.6	**1.4**	1.1	1.8	**2.2**	1.9	2.4
Age group 55-74	**2.7**	**1.9**	1.5	2.3	**2.1**	1.1	3	**1.7**	1.3	2.1	**2.4**	2	2.7
Age group 75-99	**3.1**	**1.9**	1.4	2.4	**1.9**	0.9	3	**2**	1.4	2.6	**2.1**	1.7	2.5
**Any entitlement to reimbursed medication**													
Continental Finland	**31**	**40.9**	38.9	42.9	**35.8**	33.9	37.8	**32.7**	30.7	34.7	**32.9**	31.5	34.3
Turku	**26.3**	**35**	33.5	36.4	**31.8**	30	33.5	**27.8**	26.1	29.4	**27.8**	26.9	28.7
Kainuu	**39**	**49.7**	48.2	51.1	**43.5**	41.9	45.1	**40.6**	39.1	42.2	**40.1**	39	41.1
Southern Oulu region	**39.1**	**48.3**	45.7	50.9	**43**	40.4	45.7	**38.7**	36.1	41.3	**39.3**	37.5	41.1
Oulu region	**29.8**	**37.5**	35.5	39.5	**33.3**	31.4	35.3	**29**	27.1	30.9	**29.8**	28.6	31
Men	**29.6**	**41.9**	40.7	43	**38.3**	35.5	41.1	**31**	29.7	32.3	**32.7**	31.9	33.5
Women	**32.2**	**42.6**	41.4	43.7	**34.1**	31.8	36.4	**32**	30.7	33.3	**32.7**	31.9	33.4
Age group 20-54	**15.5**	**19**	18	19.9	**18.5**	16.3	20.6	**17.1**	16	18.2	**19.1**	18.4	19.7
Age group 55-74	**48.9**	**50**	48.7	51.4	**48.1**	44.8	51.4	**49.6**	47.9	51.2	**49.2**	48.1	50.3
Age group 75-99	**75.3**	**74.5**	73	76	**74.6**	71.1	78	**74.5**	72.8	76.3	**74.6**	73.5	75.8

^*^95% confidence intervals (CI).

^§^Crude prevalences are based on participants without weighting.

^#^Sampling weighted (SW) prevalence estimates are based on participants and sampling probabilities.

^†^Inverse probability weighted (IPW) prevalence estimates are corrected for the nonresponse.

^‡^Doubly robust (DR) prevalence estimates are corrected for the nonresponse.

True prevalences based on the full sample and sampling probabilities, and corresponding estimates based on participants and different statistical methods to account for missing data in continental Finland and in different subgroups.

## Discussion

We applied the IPW method to handle both the differential sampling probabilities and missing data. Age and gender were the most important factors associated with non-response. Other register-based variables – marital status, education, area and language – were also important. The model selection procedure based on the BIC was well suited to selecting an optimal and parsimonious logistic regression model to predict the non-response. The class of candidate models did not, however, cover all possible interactions or nonlinearities. Better models could thus yet be found. However, such models might be more difficult to interpret and the effects of the predictors more difficult to understand. The BIC also avoids the problems of stepwise regression procedures and the Akaike’s information criterion; see e.g. [24]. The GBM did not markedly improve the predicted probability of response when compared with the logistic regression model selected using the BIC.

The weighting appeared to improve the accuracy of the estimates, as we demonstrated with the turnout percentages. Granberg and Holmberg [32] compared the true and self-reported turnout at the individual level. They found that 99% of the voters said that they voted but only 74% of the non-voters said that they did not vote; thus, the sensitivity was very good but specificity was not good. Assuming that this result was also true for the 2008 Finnish election, the true self-reported turnout percentage without missing data would have been 70.7%, which was within the CI of our weighted estimate. In the other study areas, the findings were similar except in Kainuu, where the turnout percentage was much lower than in continental Finland as a whole. It is plausible for non-response to be associated with low turnout in elections, in which case the turnout percentages were overestimated. This assumption is supported by Martikainen et al. [33], who found that in young age groups low education was associated with low turnout percentages, and in our study this group had the lowest participation rates. The percentage of over-reporting in our study (16.9%) based on the crude self-reported turnout was slightly lower than in the local elections in Sweden (22%) between 1988 and 1998 [34], and thus we consider that our correction [32] was based on a sensible choice. We considered the percentage of non-citizens who were eligible to vote in municipal elections but who were not included in the ATH sampling frame to be so low that it created only little bias.

The IPW and DR methods appeared to correct most of the bias in the prevalence estimates of entitlement to specially reimbursed medication for any chronic disease. However, the disease-specific estimates were almost as good when using the sampling weights indicating that the non-response was particularly selective in other diseases than diabetes, psychosis and other neurological diseases.

The inverse probability-weighted BMI estimates differed from the crude estimates in the subpopulation of people aged 20 to 59 with basic or unknown education in Turku, but the differences were smaller in other education groups and areas (data not shown). This result indicates that those people who had larger values of the weight variable had smaller BMI values. According to the results of the missing data analysis, the young, unmarried and non-Finnish-speaking men in the lowest education class had the lowest response rate and therefore also the largest weight variable values. FPC had influence on the variance estimates only in older age groups and those geographical areas where the sampling fraction was high.

There are also other methods for handling the effects of missing data. Multiple imputation [35] is the most suitable method in cases where item response rates vary considerably and the proportion of complete cases is much lower than the item response rates. This was not the case in the ATH study, and thus the weighting-based methods perform well. An alternative approach to IPW is poststratification [13], which is based on a saturated response model. In this method, the response rates are estimated separately in poststrata defined by some register-based variables, and the inverses of these response rates are then used as weights in analyses. The number of poststrata increases exponentially with the increase in the number of variables or their categories, and thus the number of sampling units per cell decreases fast. The method based on poststratification weights is thus the most suitable for a small number of poststrata. We also assessed different interactions in the weighting model using the BIC, but few interactions were found; thus poststratification would provide little improvement over the IPW method that we have applied.

It has been shown that a weighting model should contain good predictors of the outcome rather than the missingness, e.g. [36–38]. Seaman and White [37] noted that in case of many different outcome variables, inclusion of variables, which predict the outcomes well, is not possible. Our aim in the ATH study has been to provide researchers general-purpose tools (based on the IPW as weights are easy to use also for non-statisticians) to handle missing data. As the ATH study contains hundreds of variables aimed to cover a large variety of lifestyle and risk factors as well as health and other outcomes, we cannot provide optimized weights for all research questions, thus we concentrated on variables with predictive power on the missingness. Furthermore, it can be noted that methods, which have been developed to estimate the effect of a treatment in observational studies, might not be directly applicable, because in those models there is usually an arrow from treatment to outcome in the causal graph. In case of handling the effects of nonresponse, however, there is seldom an arrow from missingness to outcome. An exception could be an intervention study in which e.g. the participants are subject to the intervention but the nonparticipants are not. In population surveys such as in our case, there is practically no intervention effect. In conclusion, we considered that it is safer to apply the variable selection methods for handling missing data suggested in e.g. [37].

Further analyses should be done using register values with information concerning the health status of the respondents and non-respondents, hospital discharge, drug reimbursement, etc. This would be important for measuring the drop-out bias and selection due to possible health-related issues. Although register-based data cover the full population, there are also shortcomings in the information content. For example, important lifestyle factors such as smoking, alcohol use, nutrition and physical exercise and also cohabitation cannot be obtained from registers. The variables of the questionnaires do not necessarily match the information contents of the registers. Furthermore, there is also a considerable time lag in the availability of data from certain registers such as the drug reimbursement register.

In the present study, we demonstrated how to implement IPW using model selection and socio-demographic register data to analyze non-response mechanisms and how this method improved the accuracy of both entitlement to specially reimbursed medication and self-reported turnout prevalences considerably, even though the non-response rate was as high as in the ATH survey. Furthermore, our analyses provided insight into the structure of missing data. Several demographic factors were shown to be associated with missing data, but few interactions among them were found.

## Conclusions

Missing data analyses showed associations between non-response and several demographic factors, but few interactions improved the predictive power over the main effects model.

Accuracy of the self-reported turnout and reimbursed medication estimates improved considerably using the methods based on weighting suggesting that the weighting methods can improve the estimates of both social activity and especially health outcomes.

Register data linked with survey data can provide more accurate results than survey data alone, and the administrative registers in Finland have been shown to have good potential in improving the quality of survey analyses.
